# White kidney bean extract as a nutraceutical: effects on gut microbiota, alpha-amylase inhibition, and user experiences

**DOI:** 10.1017/gmb.2023.5

**Published:** 2023-06-01

**Authors:** David Houghton, Oliver M. Shannon, Peter I. Chater, Matthew D. Wilcox, Jeffrey P. Pearson, Kyle Stanforth, Cara Jordan, Leah Avery, Alasdair P. Blain, Abraham Joel, Ruth Jeffers, Ruth Nolan, Andrew Nelson, Christopher J. Stewart, Fiona C. Malcomson

**Affiliations:** 1 Wellcome Centre for Mitochondrial Research, Translational and Clinical Research Institute, Newcastle University, Newcastle upon Tyne, UK; 2 Human Nutrition and Exercise Research Centre, Population Health Sciences Institute, Newcastle University, Newcastle upon Tyne, UK; 3 Biosciences Institute, Newcastle University, Newcastle upon Tyne, UK; 4 School of Health and Life Sciences, Teesside University, Tees Valley, UK; 5 School of Agriculture and Food Science, University College Dublin, Dublin, Ireland; 6 Faculty of Health and Life Sciences, Northumbria University, Newcastle upon Tyne, UK; 7 Translational and Clinical Research Institute, Faculty of Medical Sciences, Newcastle University, Newcastle upon Tyne, UK

**Keywords:** Gut microbiota, inflammation, α-amylase, white kidney beans, model gut

## Abstract

White kidney bean extract (WKBE) is a nutraceutical often advocated as an anti-obesity agent. The main proposed mechanism for these effects is alpha-amylase inhibition, thereby slowing carbohydrate digestion and absorption. Thus, it is possible that WKBE could impact the gut microbiota and modulate gut health. We investigated the effects of supplementing 20 healthy adults with WKBE for 1 week in a randomised, placebo-controlled crossover trial on the composition of the gut microbiota, gastrointestinal (GI) inflammation (faecal calprotectin), GI symptoms, and stool habits. We conducted *in vitro* experiments and used a gut model system to explore potential inhibition of alpha-amylase. We gained qualitative insight into participant experiences of using WKBE via focus groups. WKBE supplementation decreased the relative abundance of *Bacteroidetes* and increased that of *Firmicutes*, however, there were no significant differences in post-intervention gut microbiota measurements between the WKBE and control. There were no significant effects on GI inflammation or symptoms related to constipation, or stool consistency or frequency. Our *in vitro* and gut model system analyses showed no effects of WKBE on alpha-amylase activity. Our findings suggest that WKBE may modulate the gut microbiota in healthy adults, however, the underlying mechanism is unlikely due to active site inhibition of alpha-amylase.

## Introduction

White kidney bean (*Phaseolus vulgaris L.*) extract (WKBE) is a popular nutraceutical, often advocated as an anti-obesity agent. As summarised in a recent review, supplementation with WKBE for up to 3 months has been shown to elicit small, but potentially beneficial, reductions in body weight in adults with overweight and obesity (1.8–3.5 kg weight loss; Nolan et al., 2020). Mechanistically, WKBE has been reported to inhibit the action of alpha-amylase (α-amylase) – an enzyme that plays a crucial role in carbohydrate metabolism by catalysing the hydrolysis of the α-(1,4) glycosidic linkages in starch and other oligosaccharides (Obiro et al., 2008) – thereby slowing carbohydrate digestion rate and absorption. In theory, such effects could have positive implications for other aspects of cardiometabolic and systemic health (Nolan et al., 2020).

The gut microbiota is a complex and diverse community of bacteria and other microorganisms that has been implicated in human health and disease (Valdes et al., 2018). Diet plays a key role in shaping the gut microbiota by providing different substrates that can impact the growth and activities of particular microbes and bacterial communities (Singh et al., 2017). By decreasing the digestion and absorption of starch, it is possible that WKBE could impact composition and metabolism of the gut microbiota and positively modulate gut health. Indeed, previous animal work has demonstrated WKBE supplementation can modulate the gut microbiota to increase markers of gut health, including higher short-chain fatty acid (SCFA) concentrations and gut barrier integrity, and reduce body weight and serum lipid levels (Monk et al., 2015; Shi et al., 2020; Avagliano et al., 2022). Only one study has explored the effects of WKBE on the gut microbiome in humans, and this randomised controlled trial (RCT) was conducted in participants with type 2 diabetes (Feng et al., 2022). There are currently no studies investigating the impact of WKBE on gastrointestinal (GI) inflammation (faecal calprotectin concentrations), GI symptoms related to constipation (e.g., abdominal pain, discomfort or bloating, or constipation), and stool consistency and frequency. User experiences also provide important insight to help inform recommendations for WKBE supplementation protocols and optimise products to meet user needs but have not been systematically explored to date.

We carried out a series of investigations to provide further insight into the effects of WKBE in the human gut in healthy individuals. We first carried out a clinical trial to explore the impact of WKBE supplementation on composition of the gut microbiota. This also provided an opportunity to explore the effects of WKBE supplementation on GI inflammation (faecal calprotectin concentrations), GI symptoms related to constipation (Patient Assessment of Constipation‐Symptoms [PAC-SYM]), and stool consistency/frequency (Bristol stool chart). Secondly, we used a series of *in vitro* experiments and a novel model gut system developed at Newcastle University to identify the optimal dose of WKBE for inhibiting α-amylase. Lastly, we gained qualitative insight into participant experiences of using WKBE via focus groups.

## Methods

### Clinical trial

#### Participant recruitment

Twenty healthy individuals aged 18–40 years were recruited through advertisements within Newcastle University. Exclusion criteria included: treatment with antibiotics or medications affecting GI function (e.g., laxatives) within 3 months prior to the study; GI disorders such as inflammatory bowel disease; currently trying to lose weight; being pregnant or lactating; unable to provide informed written consent; use of other dietary supplements such as prebiotics and probiotics; alcohol consumption over the national guidelines (>14 units per week); smoking; known allergies to any components within the dietary supplement or control; diagnosed kidney, liver or cardiovascular disease, recent history of cancer (past 1 year) or presence of diabetes. Ethical approval was obtained (reference: 1831/16861/2019) and all study procedures were conducted in accordance with the Declaration of Helsinki.

#### Study design

**Figure 1.**
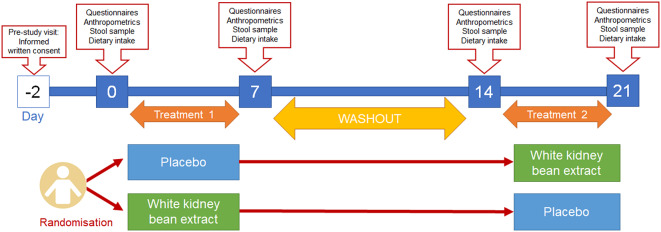
Study design.

This was a double-blind, randomised, placebo-controlled cross-over trial (Figure 1). At the “pre-study visit” on day 0, eligible and willing volunteers provided informed written consent. Participants were randomly allocated to start with intervention (WKBE) or control by a blinded member of the research team using an online randomisation tool (Urbaniak and Plous, 2013).

#### Intervention

The WKBE (Calorie Balance Ltd., UK) used in this study is a naturally derived protein found in white kidney beans. Corn starch was used as the control. Both the control and WKBE supplement were administered in capsule form, and were identical in appearance and weight. Participants were asked to take 2 × 400 mg capsules three times per day for 1 week with water before each meal. This was followed by a 1-week washout period to minimise any carryover effects of the first intervention, after which participants started their second 1-week intervention period with the alternative supplement.

#### Participant demographics

Anthropometric measurements were collected by trained research staff, including weight and body fat percentage (DC-430 MAS, Tanita Europe B.V., the Netherlands), and waist and hip circumferences measured using a tape measure and standard procedures. Height was measured using a Leicester stadiometer (Seca) in the Frankfurt plane. Body mass index (BMI) and waist:hip ratio were calculated. All measurements were performed twice or repeated until within 0.1 cm for height, 0.1 kg for weight, and 1 cm for waist and hip circumferences. The mean of the two closest measurements was used for further analyses. Measurements were made at the study visits before and after each intervention period. Dietary intake data were collected using Intake24, a validated web-based dietary recall system (Simpson et al., 2017), which yields information on energy, macronutrient and micronutrient intake, completed on three separate days (two weekdays and one weekend day) during each intervention period.

#### Stool collection

Participants were asked to collect four stool samples, that is, one before and one after each of the two intervention periods. Stool collection kits (FECOTAINER^®^, AT Medical BV, the Netherlands) were used as per the manufacturer’s instructions, and participants collected two aliquots per stool sample (one for faecal calprotectin analyses and the other for microbiota analyses), which were subsequently stored at −80˚C until further analysis. In total, 80 stool samples were analysed by all techniques.

#### Gut microbiota

DNA was extracted as previously described (Houghton et al., 2018) and detailed in the Supplementary Material. The 16S rRNA gene pipeline data incorporates phylogenetic and alignment-based approaches to maximise data resolution. The read pairs were demultiplexed based on the unique molecular barcodes, and reads were merged using USEARCH v7.0.1090 (Edgar, 2010), allowing zero mismatches and a minimum overlap of 50 bases. Merged reads were trimmed at first base with a *q* ≤ 5. In addition, a quality filter was applied to the resulting merged reads and reads containing above 0.05 expected errors were discarded. 16S rRNA gene sequences were clustered into operational taxonomic units (OTUs) at a similarity cut-off value of 97% using the UPARSE algorithm (Edgar, 2013). OTUs were mapped to an optimised version of the SILVA Database containing only the 16S V4 region to determine taxonomies (Quast et al., 2013). Abundances were recovered by mapping the demultiplexed reads to the UPARSE OTUs. A custom script constructed a rarefied OTU table from the output files generated in the previous two steps for downstream analyses of alpha-diversity, beta-diversity, and phylogenetic trends (Lozupone and Knight, 2005).

#### Gastrointestinal inflammation, symptoms, and stool frequency

Participants were asked to record self-reported symptoms as measured by the PAC-SYM questionnaire (Frank et al., 1999), as well as a Bristol stool chart (Lewis and Heaton, 1997) to assess stool frequency and consistency. Faecal samples were analysed by a hospital laboratory (Newcastle Laboratories, Royal Victoria Infirmary, UK) for the quantification of calprotectin concentrations.

### In vitro and model gut analyses

#### Alpha-amylase activity assay

Alpha-amylase activity was assessed using the 3,5-dinitrosalicylic acid (DNSA) α-amylase assay, which quantifies reducing sugar produced by α-amylase cleavage of substrate starch (Sumner and Graham, 1921). The method was followed as per Balasubramaniam et al. (2013), with slight modifications (detailed in Supplementary Material) to investigate the affect of a 1-h pre-incubation at varying pH as reported by Le Berre-Anton et al. (1997).

#### Alpha glucosidase method

The activity of the alpha-glucosidase enzyme was measured by an adapted colorimetric method using *p*-nitrophenyl-α-glucopyranoside (PNPG) as a substrate (Apostolidis et al., 2011). Alpha-glucosidase hydrolyses the PNPG to α-d-glucopyranoside and *p*-nitrophenol to produce a yellow product, which can be measured spectrophotometrically at 405 nm. The rate of colour development is directly proportional to the enzyme activity.

#### Model gut system

Model gut analysis was conducted in an artificial simulation of the upper GI tract. The system was set up and solutions prepared as previously described (Houghton et al., 2014). Analysis of carbohydrate digestion was also conducted as previously described (Chater et al., 2015). Briefly, 1 g of corn starch was used as substrate, and WKBE was tested at a dose of 800 mg to reflect an expected dose in humans. Samples were taken over a time course of 0, 30, and 60 min in the gastric phase, and 60, 75, 90, 120, 150, and 180 min in the small intestinal phase and precipitated 1:1 in 10% TCA. In order to separate products of digestion from undigested starch substrate, 50 μl of supernatant were mixed with 950 μl of 1% KCl (w/v) 75% methanol solution (v/v) and after 20 min samples were centrifuged at 9,300 rcf for 10 min. About 500 μl of the resulting supernatant were then evaporated down to a volume of 100 μl. Once cooled to 37°C, 50 ul of 1 mg/ml α-glucosidase (Sorachim) were added and incubated at 37°C for 2 h. Liberated glucose was then assayed using the Megazyme d-Glucose (glucose oxidase/peroxidase; GOPOD) Assay Kit.

### Qualitative analyses

We conducted a qualitative focus group study in accordance with the consolidated criteria for reporting qualitative research (COREQ) guidelines (Tong et al., 2007). This aimed to identify motivations for uptake of the supplement, expectations and whether participants found the supplement to be acceptable in terms of frequency of ingestion, side effects, and overall effects observed. A standardised topic guide was used to facilitate discussion and all focus group discussions were audio recorded and transcribed verbatim. Further details can be found in the Supplementary Material.

### Statistical analyses

#### Clinical trial

Potential effects of the interventions on anthropometric measurements were investigated using ANCOVA, with the post-intervention measurement as the outcome and adjusting for baseline measurement as covariate.

The analysis and visualisation of the gut microbiota data were conducted using the phyloseq package within R (McMurdie and Holmes, 2013). Sample data were imported and α- and β-diversity metrics were calculated. All samples were rarefied to 13,925 reads. Non-parametric Mann–Whitney test was used to test for significant differences within (pre vs. post) and between (WKBE vs. control) groups. Differences in beta-diversity, weighted and unweighted Unifrac were assessed using PERMANOVA (Kelly et al., 2015). All *p*-values were adjusted for multiple comparisons with the FDR algorithm (Benjamini and Hochberg, 1995).

To investigate changes in stool consistency (assessed using ROME III cut-off values) and GI symptoms (PAC-SYM data), standard chi-squared tests and original chi-squared tests were used, respectively. All analyses were corrected for multiple comparisons, where appropriate.

#### In vitro assay and model gut system

To examine the interaction between pH and group on glucose inhibition and time and group, a two-way ANOVA was performed. A linear regression was performed to study the effects of glucose release in the model gut, comparing the effects of time and group.

#### Qualitative analyses

Qualitative data were analysed using thematic analysis. All focus group transcripts were independently read and re-read by two researchers (LA and CJ); both researchers independently coded segments of data from the first transcript initially to develop a coding strategy and to generate preliminary themes. Following discussion, the same two researchers independently coded the remaining three transcripts, discussed and interpreted findings before agreeing a final set of themes that best represented the data. Disagreements were resolved by revisiting transcripts. Supporting direct quotes from participants were applied to the agreed thematic labels.

## Results

### Clinical trial

#### Study participant characteristics

Twenty participants (12 females and 8 males) completed the study and there were no adverse effects reported. Participant characteristics are summarised in Table 1. The mean age of participants was 30 years and ranged from 22 to 38 years old, and the majority (75%) were Caucasian. There were no significant differences in changes in body composition (Table 1) or in dietary intake between the WKBE and control supplementation periods (Supplementary Table S1).

**Table 1.** Participant anthropometric measurements pre- and post-supplementation with WKBE and control

	WKBE		Control				
	Pre	Post	Pre	Post	Time	Treatment	Time × treatment
Weight (kg)	64.8 (15.0)	64.6 (15.4)	65.1 (15.1)	64.6 (15.1)	0.001	0.178	0.518
BMI (kg/m^2^)	22.3 (3.1)	22.2 (3.2)	22.4 (3.1)	22.2 (3.1)	0.003	0.358	0.756
Body fat (%)	17.5 (7.0)	17.9 (7.9)	17.7 (7.7)	17.5 (7.8)	0.791	0.608	0.252
Waist circumference (cm)	75.4 (10.2)	74.8 (10.5)	76.2 (10.9)	74.9 (10.7)	<0.001	0.380	0.317

Data are presented as means and standard deviation in parentheses (SD). *P*-value for two-way repeated measures ANOVA.

#### Effects of WKBE on the gut microbiota and on markers of gastrointestinal health

Taxonomic profiles of all samples reflected a composition expected for adult human gut microbiota samples, with *Bacteroidetes* and *Firmicutes* as the dominant phyla. We compared pre- versus post-measurements (day 0 vs. day 7 of each treatment period), as well as post-intervention WKBE versus control measurements (day 7 of each treatment period) to explore changes within and between the treatment periods, respectively. When comparing pre- versus post-intervention values within the WKBE period, there was a significant change in the relative abundance of the two dominant phyla (Supplementary Figure S1). The relative abundance of *Bacteroidetes* significantly decreased and of *Firmicutes* significantly increased (*p* = 0.030) after 7 days of supplementation with WKBE (*p* = 0.030). In contrast, there were no significant changes in the relative abundance of bacterial phyla after supplementation with placebo. However, there were no significant differences in the relative abundance of dominant phyla post-intervention between the two treatment periods (Supplementary Figure S1). At the genus level, the four bacteria with the highest relative abundance were *Bacteroides*, *Faecalibacterium*, *Prevotella,* and *Alistipes* (Supplementary Figure S2). There were no apparent changes observed in the relative abundance at the genera level following each treatment period, or post-intervention differences between WKBE and control treatments (Supplementary Figure S2).

**Figure 2.**
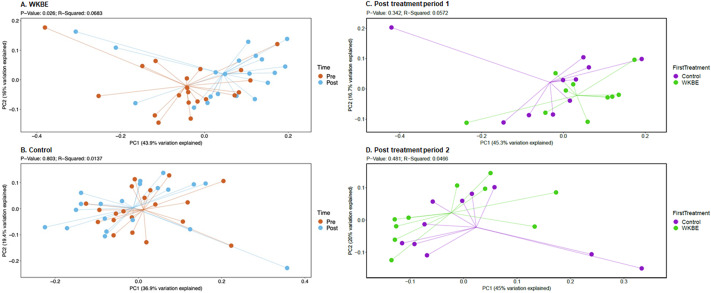
Principal component analysis of weighted UniFrac analysis comparing β-diversity pre- and post-supplementation with WKBE (A) and control (B), and post first treatment period (C) and second treatment period (D) (*N* = 20).

There were no significant changes in α-diversity, assessed by the number of OTUs and Shannon diversity, following supplementation with WKBE or with placebo, or post-intervention between the two treatment periods (Supplementary Figure S3). Beta-diversity was analysed using weighted and unweighted UniFrac analysis. Weighted UniFrac analysis showed a significant change in β-diversity following supplementation with WKBE (*p* = 0.026) but not with placebo (*p* = 0.803; Figure 2A,B). However, there were no statistically significant differences in post-intervention weighted UniFrac β-diversity between the WKBE and control treatment periods, regardless of treatment order (Figure 2C,D). There were also no statistically significant changes in β-diversity assessed using unweighted UniFrac following either intervention period (Supplementary Figure S4).

There were no significant changes in GI inflammation assessed from faecal calprotectin concentrations in either WKBE or control arms of the study (Table 2). Similarly, there were no observable changes in PAC-SYM scores (Supplementary Figure S5) or stool consistency and frequency (Supplementary Figure S6) in either WKBE or control arms of the study.

**Table 2.** Faecal calprotectin concentrations pre- and post- supplementation with WKBE and control

	WKBE		Control				
	Pre	Post	Pre	Post	Time	Treatment	Time × treatment
Faecal calprotectin (μg/g)	32.3 (53.0)	38.8 (55.8)	37.8 (62.2)	58.8 (111.3)	0.359	0.399	0.600

Data are presented as means (SD). *P*-value for two-way repeated measures ANOVA.

### In vitro and model gut analyses

#### Effect of pH on alpha-amylase activity assay

In order to investigate the effects of WKBE on alpha-amylase activity, and the potential moderating effect of pH, we attempted to replicate the experiments by Le Berre-Anton et al. (1997). With WKBE at pH 4.5, there was an apparent reduction in alpha-amylase activity of ~20% when compared with a pH 7.0 control without WKBE. However, when compared with pH-matched controls, there were no significant differences between WKBE and control at any pH (*p* > 0.05; Supplementary Figure S7).

#### Effects of WKBE in the model gut

**Figure 3.**
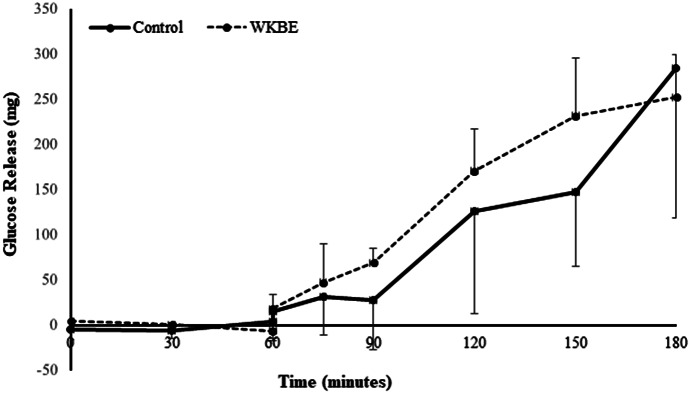
Corn starch digestion in a model gut system with control and with 800 mg white kidney bean extract (WKBE) (*n* = 6). Data are presented as means and standard error of the mean (SEM).

The digestion of 1 g corn starch substrate was tested with and without (control) 800 mg WKBE. Corn starch was digested following the expected pattern in the model gut, with minimal digestion during the gastric phase (0–60 min) and linear glucose release during the small intestine phase (60–180 min; Figure 3). However, there were no significant differences between WKBE and control between treatments at any timepoint (*p* = 0.230). The linear regression revealed a highly significant temporal effect of time (*p* < 0.001; Figure 3) on glucose release, replicating the effects expected during digestion in the GI tract.

### Participant experiences

Four focus group discussions were conducted with 18 participants (12 females and 6 males), each lasting an average of 47 min. Thematic analysis generated seven themes, presented in Table 3 alongside a description of each theme with supporting direct quotes.

**Table 3.** Themes generated from qualitative focus group discussions, a description of each theme and supporting direct quotes

Theme	Theme description	Supporting direct quotes
Expected positive impact on gut health and weight regulation	Uptake of the supplement was largely driven by the expectation that it would have health benefits. Specifically, benefits on gut health and weight loss. It was reported that ageing makes weight regulation difficult and this impacts on body image and self-esteem. If a supplement could promote weight regulation, that was desirable, although it was generally acknowledged that the duration of the intervention would limit effects on weight. Linked to outcomes, when participants were asked whether they would recommend the supplement to others, there was a consensus that they would if it led to desirable outcomes, and that these hadn’t yet been realised	“I just think any kind of health-related things like sleep or gut-micro or anything like that it really” (FG 2, P1, Male) “Mine [incentive] was financial and potential weight loss” (FG 1, P5, Female) “For me I think it was to check if it actually affects weight loss.” (FG 1, P4, Male) “It’s a short period of time, in terms of weight loss, maybe not in a short period of time, but you expect some changes.” (FG 1, P1, Male) “I think if the results came back really positive I would probably recommend it to people” (FG 1, P6, Female)
Gut health and exercise performance	Participants expressed the view that gut health plays an important role in overall health, and in some cases exercise performance. Reference was made to the growing evidence that links gut health to improved performance in endurance sports, and as such this was reported as an incentive to taking part	“I know gut health is important for just about every aspect of health related to the body…. so I was really interested in making sure that everything else was good.” (FG 1, P3, Male) “I’d just say having a healthy gut is as important as having anything else healthy in your body.” – P2 (FG 3, P2, Male) “From an exercise performance perspective as well, because there seems to be increasing evidence in links between better gut health and I guess performing better in endurance sports” (FG 1, P6, Female)
Supplementation to optimise diet and improve gut health	Supplementation was viewed as a positive approach to optimising diet, and an easy way to target gut health, without the need to make drastic changes to diet, specifically when this could impact family members. A minority of participants referred to how their gut health has changed with ageing, and how they hoped the supplement could help with this	“If it took care of all the gut health side of things, then that could be an easy way to address that and not really have to think about it” P6 (FG 1, P6, Female) “Having something as easy as a pill that is going to do the job” (FG 1, P4, Male) “It would be good for a family, way to sort of maintain gut health without having to drastically change a child’s diet” (FG 1, P5, Female)
Duration of supplementation and outcome expectations	Participants reported feeling sceptical about the likely benefits of the supplement due to the requirement to take it for such a short duration. A minority of participants referred to the little evidence available to support the effectiveness of such an intervention, and these beliefs impacted outcome expectations. Noteworthy, one participant referred to the supplement consisting of bean extract, and therefore expressed concerns about side effects, in particular bloating. In this regard the suitability of the supplement for people with irritable bowel syndrome was raised	“It is a short period of time … in terms of weight loss, maybe not in a short period of time, but you expect some changes. Gut health I was sitting on the fence whether it would work or not” (FG 1, P1, Male) “Functional outcomes like the other inflammatory markers or bowel habit and what was the other one … term weight loss would probably be too short to really see an effect” (FG 1, P2, Female) “I thought it would have taken longer to have an effect than what we were involved in. I would have thought 3 to 6 months maybe just to build up long lasting effect.” (FG 4, P1, Female)
Supplement frequency, eating habits, and adherence	The frequency of taking the supplement (three times per day) was reported to be acceptable, however for a minority of those who did not eat three meals per day, it was considered a barrier to adherence and long-term use of the supplement. One participant reported a tendency to skip breakfast, however this was reconsidered due to the requirement to take the supplement ahead of each meal, three times per day. Although pairing the supplement with meals was considered a facilitator to adherence, participants also reported the need to develop strategies to prompt them to take it. There was consensus that the supplement should be taken as part of a healthy eating regime and if it is to be marketed for mainstream use, this message should be emphasised. The capsules were considered large in size and there was the suggestion that this could be a barrier to others from taking it	“I’m the kind of person that doesn’t eat meals when I’m not hungry, sometimes I skip breakfast…. I had to force myself to have 3 meals to be able to take the supplement.” (FG 2, P5, Female) “I had to leave mine on my desk at work so they were in front of me whilst I was eating.” (FG1, P1, Male) “Yeah, just before each meal, I had them by the kettle in the morning and others I took to work with my lunch” (FG 3, P2, Male) “The word supplement stands out for me, it’s not there to replace its there to compliment. There’s no such thing as a quick fix…. I think it needs to be in conjunction” (FG 1, P5, Female)
Natural sources provide reassurance about safety	The safety of taking supplements was discussed consistently and expectations were that the supplement was made from natural sources. On that basis, confidence was reported about the safety of the supplement, and that was linked to the likelihood of long-term use should it be effective	“I think so because it was more natural as well it was more of a good thing, rather than me thinking I was taking something you know a bit questionable” (FG 1, P1, Male) “It’s just a natural product you’re not going to worry about negative effects it might be having” (FG 1, P5, Female)
Long-term use associated with effectiveness	Expectations of the effectiveness of the supplement were limited based on the short duration of the intervention time-period, however participants consistently reported a willingness to take the supplement long-term if it had observable benefits and consistent evidence of effectiveness	“I think as long as it has shown somewhere that is has worked and it is quite hard evidence that would be fine for me” (FG 1, P2, Female) “Claims that a supplement makes you lose weight… (FG 2, P3, Male) “There would have to be evidence to help support the long-term benefits.” (FG 4, P1, Female)

FG, focus group; P, participant.

## Discussion

This study applied a mixed-methods approach to explore the effects of WKBE on the composition of the gut microbiota, markers of GI health, and user preferences. Although we found that WKBE supplementation for 7 days significantly decreased the relative abundance of *Bacteroidetes* and increased the relative abundance of *Firmicutes*, there were no statistically significant differences in post-intervention levels of these bacterial phyla between the WKBE and control treatment periods. Only one other study has explored the effects of WKBE on the gut microbiota in humans (Feng et al., 2022). In 55 participants randomised to consume 1.5 mg WKBE or placebo (maltodextrin) half an hour before each meal for up to 4 months, there were significant differences in beta-diversity, but not alpha-diversity, between individuals in the WKBE and placebo group (Feng et al., 2022). After 4 months intervention, participants in the WKBE group had a significantly higher abundance of *Bifidobacterium* and *Adlercreutzia,* and lower abundance of bacteria including *Citrobacter* and *Cronobacter*, as well as greater improvements in metabolic outcomes such as HbA1c concentrations and fasting and post-prandial glucose levels (Feng et al., 2022). As the effects of WKBE were attenuated after 4 months intervention compared with after 2 months, the authors concluded that this was likely due to gradual diminished improvements to the gut microbiota (Feng et al., 2022), suggesting that supplementation duration may be important. Most of the evidence in relation to the potential effects of WKBE on the gut microbiota comes from a small number of studies in animal models which suggest beneficial effects on gut microbiota composition and SCFA concentrations (Song et al., 2016; Neil et al., 2019; Shi et al., 2020; Wang et al., 2021; Avagliano et al., 2022). Most of these have explored the role of the gut microbiota as an underlying mechanism for the effects of WKBE in models of obesity or metabolic syndrome and are summarised in our previous review (Nolan et al., 2020). For example, in C57BL/6J mice fed a high-fat diet supplemented with WKBE, WKBE increased the relative abundance of the phyla *Verrucomicrobia* and *Actinobacteria*, associated with protective effects against weight gain and hyperlipidaemia, and increased the relative abundance of the genre *Bifidobacterium, Lactobacillus,* and *Akkermansia*, associated with protective effects against obesity and metabolic disorders (Song et al., 2016). In another study investigating the anti-obesogenic activity of WKBE in a polygenic mouse model of obesity, WKBE significantly increased concentrations of total bacteria and of *Akkermansia muciniphila*, a species which has been reported to be inversely associated with obesity, diabetes and inflammation, and reduced the ratio of *Firmicutes* to *Bacteroidetes* (Neil et al., 2019). More recently, Wang et al. explored the effects of WKBE as a food additive on host metabolism in hyperglycaemic mice and reported an increase in the richness and abundance of microbial species following feeding of mice with a yoghurt containing WKBE (Wang et al., 2021). They also reported differences in *genus* related to host metabolism, including *Prevotella, Bacteroides,* and *Ruminococcus*, which have all been associated with the regulation of metabolism.

Based around our observed effects of WKBE on the gut microbiota, we might anticipate changes in α-amylase activity, as the bacteria that were modulated by WKBE are those primarily involved in the digestion of starch and dietary fibre. Such inhibition of starch and fibre digestion in the upper GI tract would provide greater amounts of fibre for digestion by bacteria in the lower GI tract. However, our *in vitro* analyses showed no differences in α-amylase activity at any pH, despite previous suggestions that WKBE is able to inhibit α-amylase through a mixed non-competitive inhibition mechanism (Le Berre-Anton et al., 1997). Therefore, our findings suggest that the underlying mechanism resulting in changes in gut microbiota composition is unlikely due to active site inhibition. The *in vitro* model gut system used only models the upper digestive tract and, due to the complexity of the large intestinal microbiota, it was decided that effects of WKBE on colonic health would be investigated *in vivo.* Potential alternative underlying mechanisms responsible for observed effects on the gut microbiota could be changes in viscosity, that is, a gel formation reducing the ability of α-amylase to digest carbohydrates, or modulation of the pH in the GI tract, thus affecting optimal pH for enzymatic activity, which has previously been reported in pectins and alginates (Chater et al., 2015; Houghton et al., 2015). Although we cannot rule out any enzymatic interactions, changes in viscosity and/or pH could also modulate starch/fibre digestion and thus also influence the gut microbiota composition, but mechanistic studies are required to elucidate further.

We did not observe effects of supplementation with WKBE on GI markers of gut health including inflammation (faecal calprotectin concentrations), stool frequency and consistency, and GI symptoms. This may be due to the sensitivity of our gut health markers. Furthermore, the selected cohort studied (healthy individuals), and duration of this study, may also have limited the impact of the WKBE on the gut health markers. Long-term studies are required to substantiate the findings presented here and to ascertain if changes in the gut microbiota due to WKBE supplementation are able to translate into clinical outcomes.

In addition to our experimental analyses, we held focus group discussions with the aim of gaining qualitative insights into the user expectations and experiences with regards to supplementation with WKBE. There was a consensus that supplements should not be used as a replacement to healthy diets, but rather be promoted as part of a healthy lifestyle. Overall, participants were positive about the source of the supplement being natural and this was reported be a facilitator to initial uptake and long-term use should it demonstrate to be effective. Expectations of the supplement included its ability to regulate gut health and weight, although it was generally accepted that this was unlikely to occur within the timeline of the study. Participants recommended changes to the supplement to maximise adherence, including smaller capsule sizes and reduced frequency of taking (i.e., one capsule per day rather than three). A consistent finding across all focus groups was the need for participants to see direct benefits from taking the WKBE supplement or to receive clear evidence of its effectiveness to promote long-term use. As the participants were knowledgeable about the benefits of gut health and were healthy, replicating this in a clinical population where weight or gut health is an issue may be beneficial and could impact on perceptions of the supplement not identified in this study.

Our mixed-method approach combined a clinical trial, *in vitro* investigations, and focus groups to provide in-depth insight into effects, mechanisms of action, and user experience of WKBE supplementation. Nonetheless, this was a small study with only 20 participants, and each intervention period lasted only 7 days. Previous studies, though these did not assess gut-related outcomes, have ranged from 28 to 90 days in length (Nolan et al., 2020), and a recent RCT investigating effects on the gut microbiota supplemented participants with WKBE for up to 4 months (Feng et al., 2022). Larger, longer studies are therefore needed to substantiate these findings. Future studies may also wish to explore the impact of WKBE on endpoints which are responsive to short-term interventions such as inflammatory cytokines and chemokines, and SCFAs – the concentrations of which might be anticipated to change following the modification to the gut microbiota.

## Conclusions and future directions

In conclusion, although we did not observe differences in the gut microbiota between the WKBE and control treatment periods, after supplementation with WKBE for 7 days there appears to be a shift in the gut microbiota towards an increase in *Firmicutes* and a decrease in *Bacteroidota.* Longer-term, large-scale investigations are warranted to confirm these findings and identify any potential implications for health. Our results also highlight the need for further research into the mechanisms through which WKBE might reduce starch digestion and modulate health, given the absence of α-amylase inhibitory activity observed during our *in vitro* investigations.

## Data Availability

Data are available upon request. For the purpose of open access, the author has applied a CC BY public copyright licence to any author accepted manuscript version arising from this submission.

## Supplementary material

For supplementary material accompanying this paper visit https://doi.org/10.1017/gmb.2023.5. click here to view supplementary material
