# Supplementary material for: White kidney bean extract as a nutraceutical: effects on gut microbiota, alpha-amylase inhibition, and user experiences
Source: Gut Microbiome (Camb). 2023 Jun 1;4:e8. doi: 10.1017/gmb.2023.5 (PMC11406411; doi:10.1017/gmb.2023.5)
Supplement: Supplementary file 1 [file S2632289723000051sup001.docx]

**SUPPLEMENTARY MATERIAL**

**SUPPLEMENTARY METHODS**

**Gut Microbiota Analysis**

DNA was extracted from 350mg of stool using the Powerlyzer Powersoil DNA isolation kit (MoBio) as per the manufactures’ protocol. Polymerase chain reaction (PCR) was used to amplify the 16S rDNA V4 region and sequenced in the MiSeq platform (Illumina) using the 2 × 250 bp paired-end protocol yielding pair-end reads that overlap almost completely. The primers used for amplification contain adapters for MiSeq sequencing and single-end barcodes allowing pooling and direct sequencing of PCR products(1).

***In vitro* Analysis**

Alpha-amylase cleavage of the starch generates reducing sugars which, when heated, reduce 3,5-Dinitrosalycylic acid to 3-amino, 5-nitrosalycylic acid to form a red coloured product. Alpha-amylase was prepared at 0.5mg/ml in 4mM Acetate buffer and titrated to appropriate pH, WKBE was made up at 2mg/ml in acetate buffer and titrated to appropriate pH. Tested pH values were pH 4, 4.5, 5, 5.5, 6, 6.5 and 7. Thirty microliters of α-amylase were preincubated for 1 hour with 30µL WKBE, or 30µL pH-matched acetate buffer as control. After a 1 hour pre-incubation, 50µL starch solution (0.5% I 40mM sorensens phosphate, pH7) were added and the plate was incubated at 37°C for 30 minutes. After 30 minutes, 60µL DNS reagent were added and the plate incubated at 70°C for 15 minutes for colour development. Change in absorption can was detected at 550nm in a 96-well plate spectrophotometer.

In order to separate products of digestion from undigested starch substrate, 50µl of supernatant were mixed with 950µl of 1%KCl (w/v) 75% methanol solution (v/v) and after 20 minutes samples were centrifuged at 9,300 rcf for 10 minutes. 500µl of the resulting supernatant were then evaporated down to a volume of 100µl. Once cooled to 37°C, 50ul of 1mg/ml α-glucosidase (Sorachim) were added and incubated at 37°C for 2 hours. Liberated glucose was then assayed using the Megazyme D-Glucose (glucose oxidase/peroxidase; GOPOD) Assay Kit.

**Focus Group**

All participants who took part in the intervention were invited to take part in a focus group discussion scheduled to take place following completion of both treatment arms. Focus group discussions were facilitated by two members of the research team (LA and CJ) using a semi-structured topic guide. Topics included motivations for taking part, previous strategies used to improve gut health or regulate weight, expectations of the supplement, and experiences using it, and views on long term use. At the beginning of each focus group discussion facilitators explained the aims and how data generated would be used. All focus group discussions were conducted remotely via video call, audio recorded and transcribed verbatim.

**SUPPLEMENTARY RESULTS**

**Table S1. Dietary intake during WKBE and Control arms of the study.**

| **Variable** | **WKBE** | **Control** | **Difference** | **P value** |
| --- | --- | --- | --- | --- |
| **Energy (kcals)** | 2196 (733:5353) | 1952 (823:3922) | 243 (-202:690) | 0.67 |
| **Fat (g)** | 84 (32:302) | 76 (32:197) | 8.1 (-21.2:37.3) | 0.81 |
| **Saturated Fat (g)** | 32 (11:131) | 30 (8:94) | 2.7 (-10.9:16.3) | 0.81 |
| **Protein (g)** | 98 (38:330) | 90 (41:164) | 8.1 (-27.7:44.0) | 0.81 |
| **Carbohydrate (g)** | 287 (60:514) | 237 (60:514) | 51 (-9:111) | 0.67 |
| **Total Sugars (g)** | 113 (22:265) | 100 (11:289) | 14 (-12:40) | 0.67 |
| **Non-Milk Extrinsic Sugars (g)** | 59 (15:148) | 52 (2:143) | 8 (-9:25) | 0.67 |
| **Alcohol (units per week)** | 6.8 (0:48) | 3.2 (0:21) | 3.7 (-1.4:8.7) | 0.67 |
| **Fibre (g)** | 17 (5:40) | 17 (4:37) | -0.1 (-4.4:4.3) | 0.98 |
| **Glucose (g)** | 18 (3:61) | 15 (3:57) | 2.6 (-5.2:10.4) | 0.81 |
| **Starch (g)** | 162 (17:514) | 133 (49:431) | 30 (-17:75) | 0.67 |

Data are presented as mean (minimum:maximum) intake per day. Difference represents the mean differences between WKBE and control arms, with 95% confidence intervals in brackets. P values are adjusted for multiple comparisons.


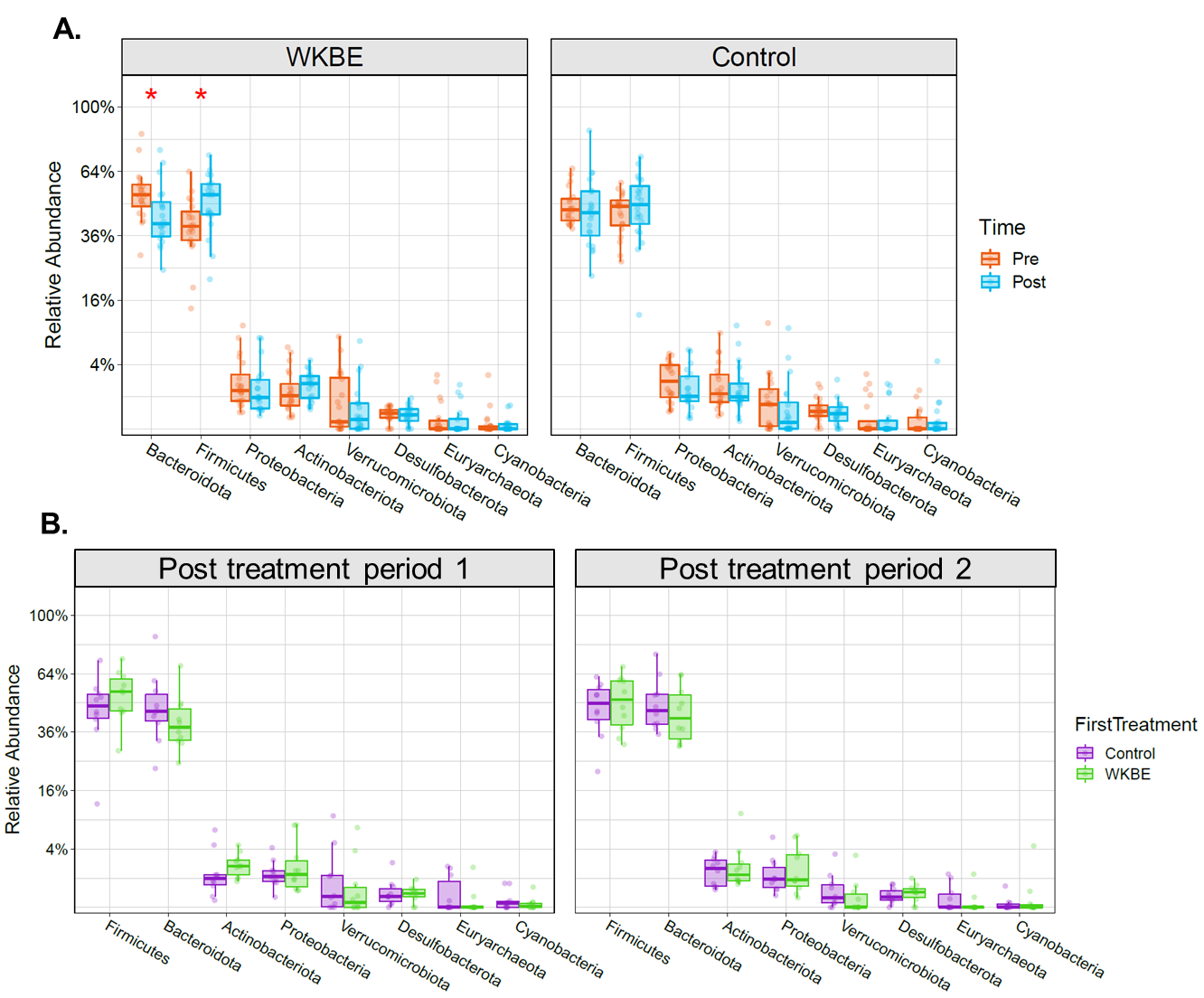


**Figure S1. Boxplots showing the relative abundance of bacterial phyla pre- and post- seven days supplementation with WKBE and Control (A) and post-intervention following each treatment period (B) (n=20).**

*denotes a statistically significant difference, Kruskal–Wallis (p<0.05).


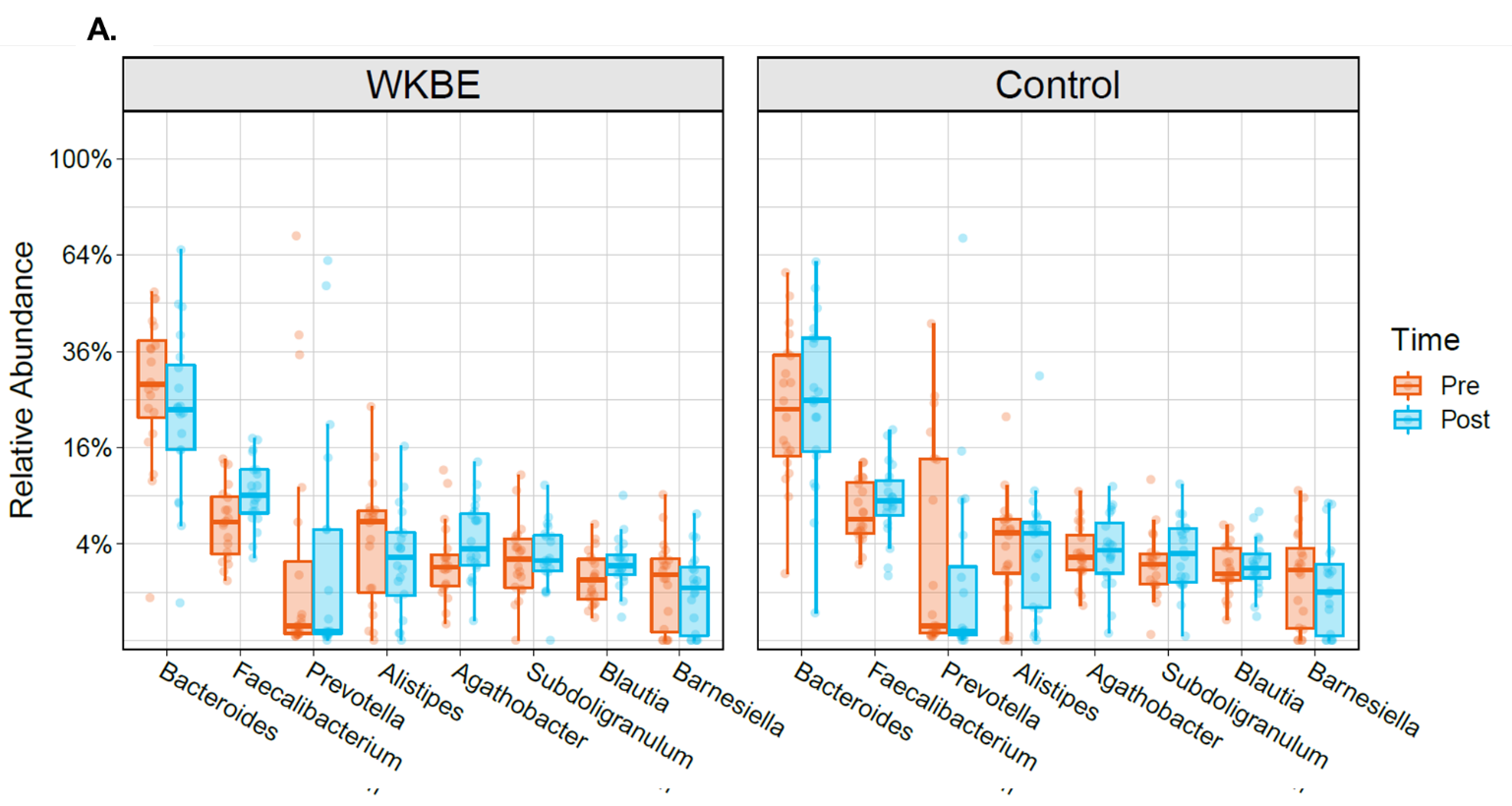


**
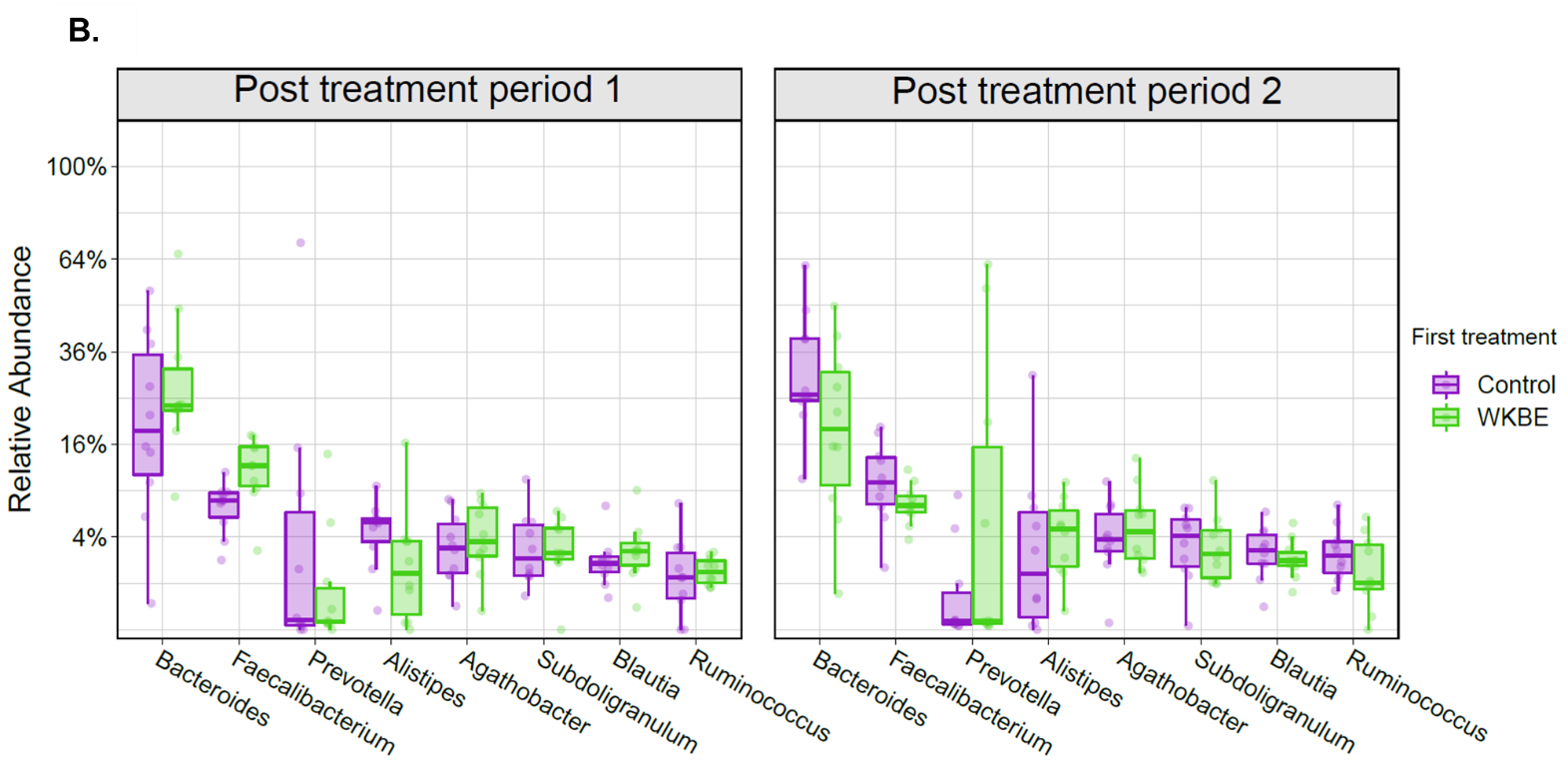
**

**Figure S2. Boxplots showing the relative abundance of bacterial phyla pre- and post- seven days supplementation with WKBE and Control (A) and post-intervention following each treatment period (B) (n=20).**


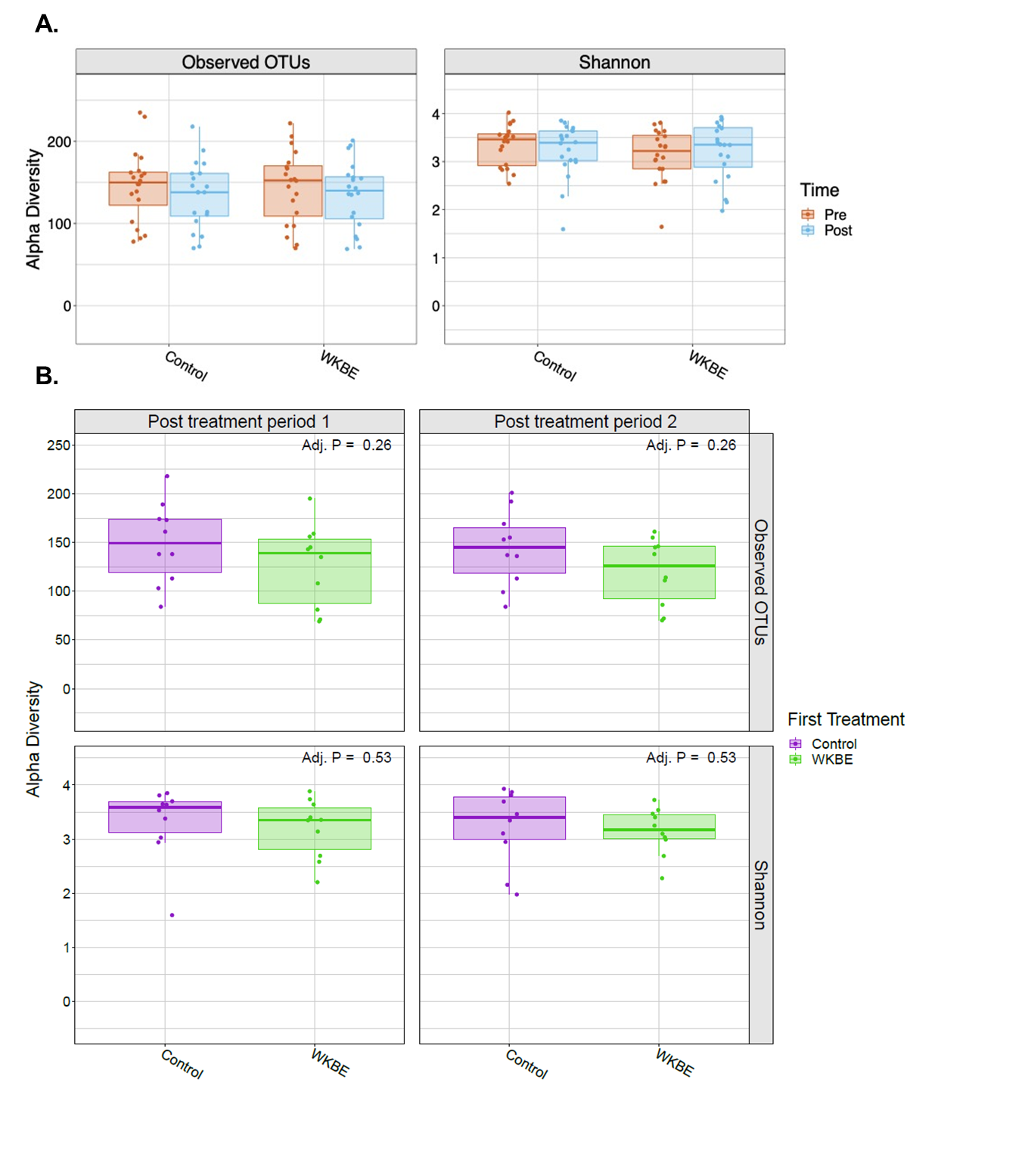


**Figure S3. Boxplots showing alpha diversity differences for observed operational taxonomic units (OTUs) and Shannon Index pre- and post- seven days supplementation with WKBE and Control (A) and post-intervention following each treatment period (B) (n=20).**

Data are presented as means and interquartile range .

**
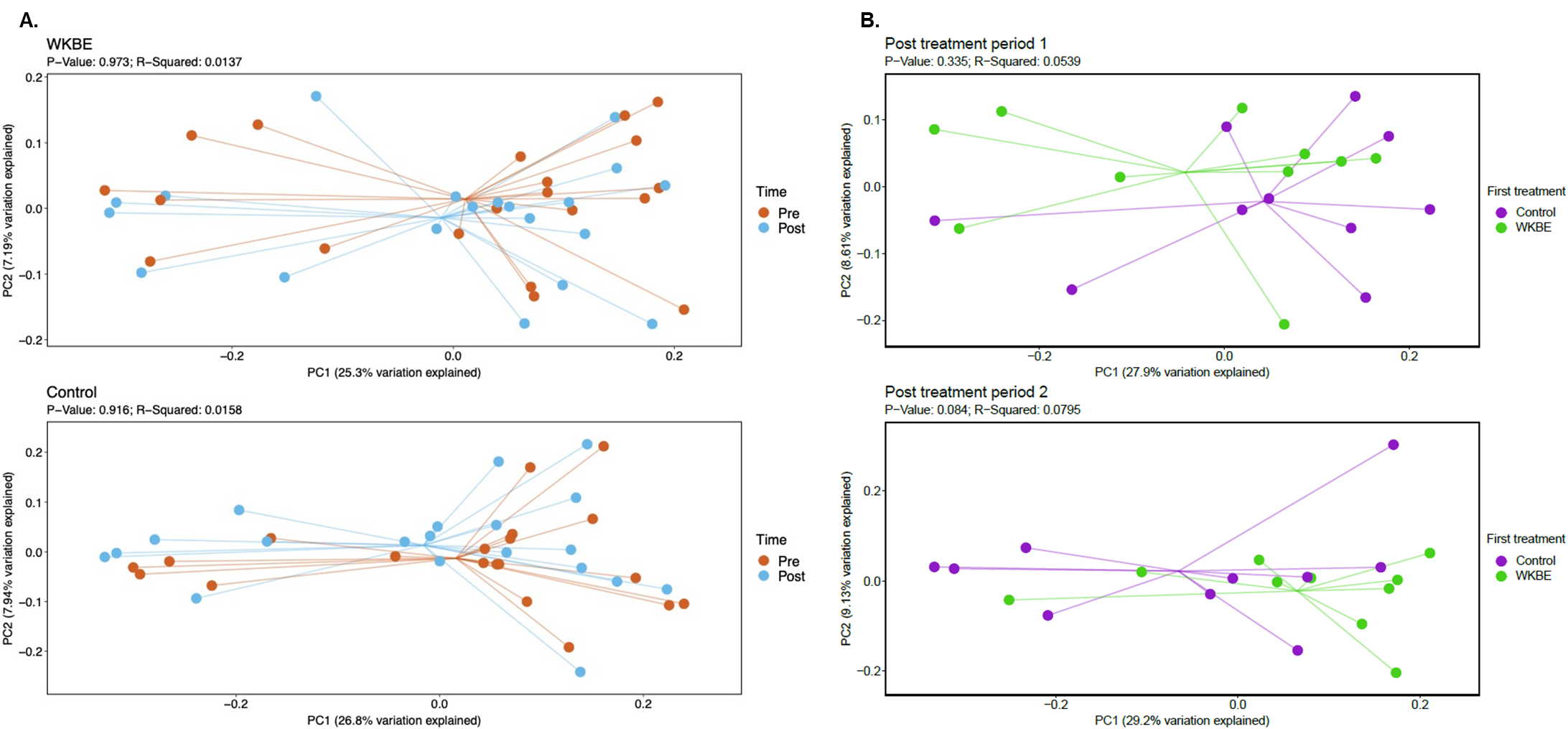
**

**Figure S4. Principle component analysis of Unweighted UniFrac analysis comparing beta-diversity pre- and post- seven days supplementation with WKBE (A) and Control (B) (n=20).**

**Figure S5: Proportional changes in Patient Assessment of Constipation Symptoms (PAC-SYM) pre- and post- seven days supplementation with WKBE (A) and Control (B) (n=20).**

**Figure S6. Dot plot of Bristol Stool Score (BSS) and stool frequency. Time points are coloured with red (WKBE) and black (Control) following each arm of the trial. The size of each point is relative to stool frequency. BSS 1-2: constipation, 3-5: normal stool, and 6-7: loose stools (n=20).**

**Figure S7. Alpha-amylase activity for WKBE and a control following incubation for 1-hour at varying pH (n = 11).**

Data are presented as mean and standard error of the mean (S.E.M)

**References:**

1. Caporaso JG, Lauber CL, Walters WA, Berg-Lyons D, Huntley J, Fierer N, et al. Ultra-high-throughput microbial community analysis on the Illumina HiSeq and MiSeq platforms. ISME J. 2012;6(8):1621-4.
